# Clinical and microbiological effects of a propolis toothpaste in patients with periodontitis under supportive periodontal therapy: a randomized double-blind clinical trial

**DOI:** 10.1007/s00784-025-06456-5

**Published:** 2025-07-09

**Authors:** Kazu Takeuchi-Hatanaka, Masahiro Ito, Yoshihiro Hayashi, Hiroe Maruyama, Hiroyuki Kono, Yuki Shinoda-Ito, Kazuhiro Omori, Shogo Takashiba

**Affiliations:** 1 Department of Periodontics and Endodontics, Okayama University Hospital, 2-5-1 Shikata-cho, Kita-ku, Okayama, 700-8558 Japan; 2 Shiomi dental clinic, 3-12-13 Kuzuhamisaki, Hirakata, 573-1112 Japan; 3 Nagaragawa Research Center, API Co., Ltd, 692-3 Nagara, Gifu, 502-0071 Japan; 4 Department of Pathophysiology–Periodontal Science, Faculty of Medicine, Dentistry and Pharmaceutical Sciences, Okayama University, 2-5-1 Shikata- cho, Kita-ku, Okayama, 700-8525 Japan

**Keywords:** Propolis, Toothpaste, Periodontitis, Periodontal pocket, Saliva, Randomized controlled trial

## Abstract

**Objectives:**

Propolis possesses antibacterial, anti-inflammatory, and antioxidant properties. While its application in oral care has garnered significant attention, evidence supporting its effectiveness against periodontal bacteria is limited. This study used a randomized double-blind protocol to assess the safety and efficacy of toothpaste containing propolis compared to a placebo in patients undergoing supportive periodontal therapy (SPT).

**Materials and methods:**

Thirty-two participants in SPT were randomized into two groups: toothpaste containing 2.5% ethanol-extracted propolis (EEP) and a placebo without EEP. Participants brushed twice daily for four weeks, and clinical parameters, bacterial counts, and salivary characteristics were assessed before and after the intervention.

**Results:**

The propolis group showed a significant reduction in periodontal pocket depth (*P* = 0.006), with a mean depth of 3.80 mm compared to 4.35 mm in the placebo group. Bleeding on probing was significantly reduced in both groups (*P* = 0.032 in the propolis group and 0.0498 in the placebo group), but did not differ between groups. Total bacterial and *Porphyromonas gingivalis* (*P. gingivalis*) counts did not differ significantly between the groups; however, the number of patients with decreased *P. gingivalis* was slightly larger than those in the placebo group (not significant). Additionally, saliva acidity decreased significantly in the propolis group (*P* = 0.041), suggesting a shift toward a less pathogenic oral environment. No adverse events were observed.

**Conclusion:**

These findings suggest that propolis may contribute to stabilizing periodontal disease during supportive periodontal therapy by modulating salivary acidity.

**Clinical relevance:**

Periodontal pocket depth and the rate of bleeding on probing are reduced, along with decreased saliva acidity. Meanwhile, the levels of *P. gingivalis* in the periodontal pockets remain low. Propolis-dentifrice may help alleviate gingival inflammation during SPT.

**Clinical trial registration:**

Registered in the University Hospital Medical Information Network Clinical Trial Registry (ID: UMIN000029554).

**Graphical abstract:**

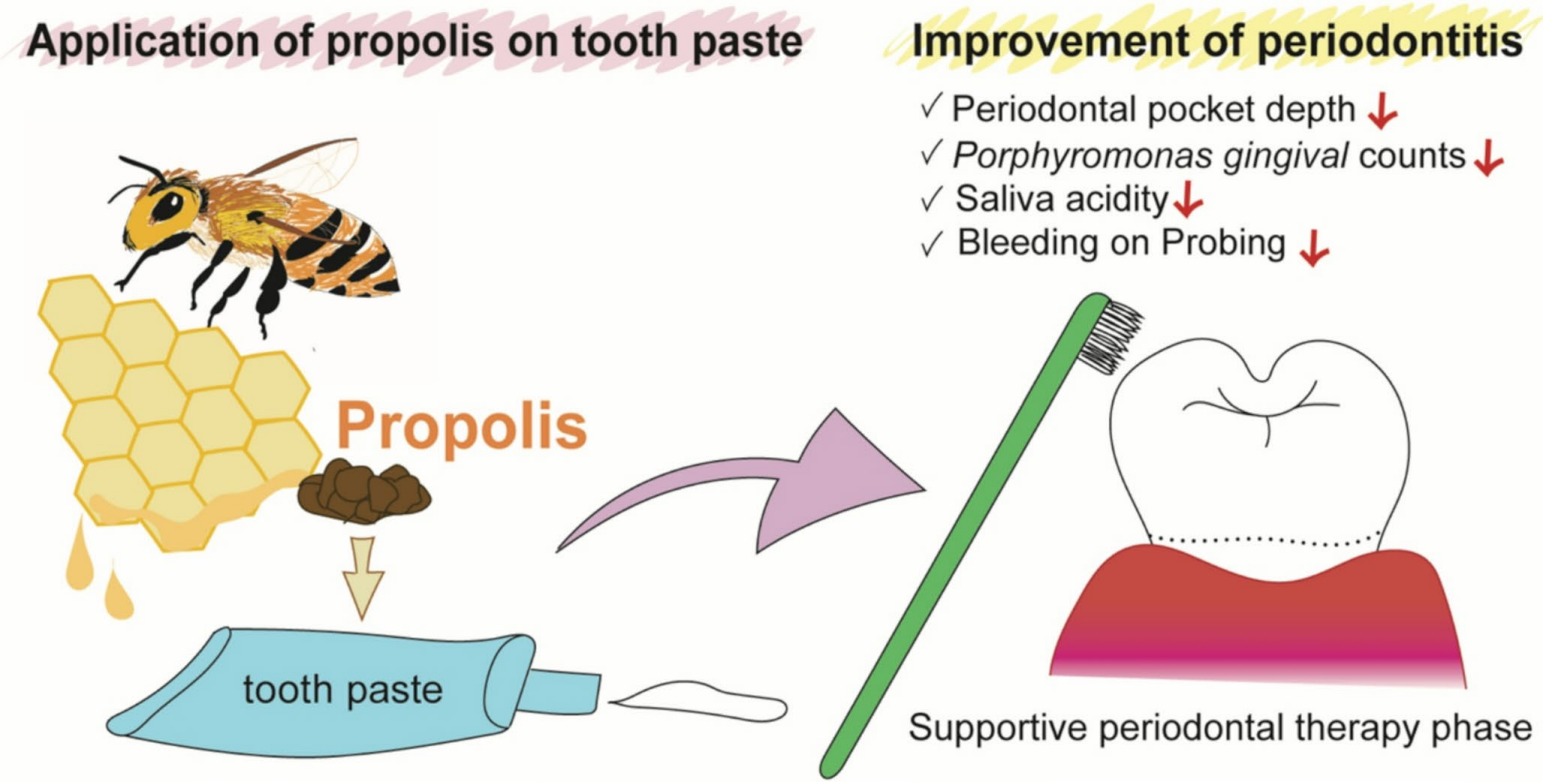

## Introduction

In recent years, natural remedies have gained preference over synthetic drugs for the treatment of periodontal disease [1, 2], and oral care products containing honeybee-derived ingredients have been developed. Propolis is a natural nontoxic resin compound produced by honeybees. Its chemical composition includes 50% resin and vegetable balsam, 30% beeswax, 5% pollen, 10% essential and aromatic oils, and other organic compounds [3–5]. Propolis has been reported to exhibit various bioactivities, including antioxidant, anticancer, antibacterial, anti-inflammatory, and anti-fungal activities [6–8]. However, it may still cause adverse effects, despite being a natural product [9–12].

Clinical trials and experimental evidence have indicated an increasing trend toward the use of natural therapies with pharmacological activity in the treatment of various oral bacterial diseases [13]. However, to date, clinical evidence validating their application to periodontal bacteria remains limited. Natural products have been utilized in various oral care formulations, including mouthwashes [14–16], gels [17–19], ointments for topical administration [20, 21], oral capsules [22], and toothpastes [19, 23, 24]. Propolis, which is the focus of this study, is produced in hives by mixing sap collected from honeybees with their saliva. It has demonstrated bactericidal, anti-inflammatory, and antioxidant effects, with several studies reporting its application in oral care products such as toothpaste [19, 25, 26]. Only one study performed a randomized-controlled trial to evaluate oral microbiota in student volunteers aged 18–40, assessing the gingival index and plaque index in individuals with an unknown condition of periodontitis [24]. No randomized double-blind clinical trials have ever compared the bacteriological and clinical efficacy of propolis in patients with periodontitis.

Therefore, this study aimed to investigate the effects of a propolis-containing toothpaste on periodontitis in patients with stable periodontal conditions undergoing supportive periodontal therapy compared to a placebo toothpaste without propolis in a randomized, double-blind trial. Bacteriological evaluation was performed to quantify periodontal bacteria and ascertain oral bacteria, including cariogenic bacteria, to determine the safety and efficacy of the toothpaste.

## Materials and methods

### Propolis-containing toothpaste

Green propolis was obtained from Minas Gerais, Brazil. The main botanical source is *Baccharis dracunculifolia* DC [27–29]. Propolis was extracted using 95% ethanol and stirred at room temperature. The insoluble part was removed by filtration and adjusted to approximately 20% solid content, designated as ethanol-extracted propolis (EEP: EEP-B20E, API Co., Ltd., Gifu, Japan). Toothpaste containing 2.5% EEP and a placebo toothpaste without EEP were prepared (API). Toothpaste containers were assigned a number to ensure unbiased allocation.

### Study population

Japanese patients with stable periodontitis who had completed active periodontal treatment were recruited from the Department of Periodontics and Endodontics at the Okayama University Hospital. They were included in this study based on the following inclusion and exclusion criteria:

#### Inclusion criteria

1. Patients regularly visit the hospital for supportive periodontal therapy (SPT) at intervals of two months or more.
2. Patients with good oral hygiene were considered capable of following oral care instructions.
3. Patients aged 20 years or older at the time of consent.
4. Patients with at least 20 remaining teeth.
5. Patients with at least three teeth presenting with 4–6 mm residual periodontal pockets.
6. Patients who provided written informed consent.

#### Exclusion criteria

1. Patients with allergies to toothpaste ingredients.
2. Pregnant or lactating females.
3. Patients with unstable systemic conditions.
4. Patients who received local or systemic antibiotics or anti-inflammatory drugs within the past three months.
5. Patients with acute periodontitis symptoms within the past three months.
6. Smokers.

The sample size was estimated based on a previous study [30, 31] and adjusted to achieve a power of 80% with a significance level of 0.05. It was determined based on the difference between groups using the population means of the two groups of plaque indices [30]. The calculation resulted in a sample size of 14 patients, with 7 in each group. Additionally, the sample size calculation for the chi-square test from the between-group comparison of BOP improvement [31] yielded 32 subjects, with 16 in each group. However, because of the absence of previous randomized clinical trials on toothpaste containing propolis, it was not possible to calculate the sample size based on bacterial counts. Considering this limitation, the minimum number of patients was estimated to be 24, with 12 in each group.

### Clinical protocol

This was a randomized, placebo-controlled, double-blind, parallel-group trial with a 1:1 allocation ratio. The trial protocol was approved by the Okayama University Ethics Committee (Clinical Research 1701-007) and registered in the University Hospital Medical Information Network Clinical Trial Registry (ID: UMIN000029554). Written informed consent was obtained from all participants after they were fully informed of the study procedure. The test toothpastes were pre-assigned, coded randomly, and distributed by designated personnel as the participants were enrolled in the study.

One of the investigators, M.I., created a simple random allocation table using a random number table prior to the commencement of the study. It was concealed until each participant’s study was completed and analysis was initiated. This approach ensured that neither the participants nor the evaluators were aware of the group assignments during the trial, maintaining the integrity of the double-blind design.

The participants brushed their teeth twice daily, in the morning and evening, for four weeks using a distributed toothbrush (Butler #211, Sunstar, Osaka, Japan) and an unreleased toothpaste. The toothpaste (Table 1) was packed at 80 g per tube and stored at room temperature. The ingredients of the control toothpaste were regular formulas used in standard toothpastes, whereas those of the test toothpaste contained regular formulas plus EEP (25 mg/g, equivalent to 0.5% propolis solid). The amount of toothpaste used was approximately two-thirds of the head length of the distributed toothbrush. The use of interdental brushes, dental floss, and other cleaning tools was not restricted. However, mouthwashes, gels, periodontal medications, and other products with potential medicinal benefits were prohibited during the study period. Bacteriological, clinical, and salivary examinations were conducted at the beginning and end of the four-week study. At the end of this study, all pastes were collected from the participants and inspected visually and by smell to check for any property changes. No chemical analysis was performed.

**Table 1:** Summary of test and control toothpaste formulations

Ingredients		Materials
Functional ingredients	Test	Propolis extract (Brazil)
	Control	None
Abrasives		calcium carbonate
Wetting agent		glycerin
		sorbitol
Binder		xanthan gum
		carrageenan
Foaming agent		soap base
Sweetening agents		xylitol
		dipotassium glycyrrhizate
Flavoring agent		peppermint oil
Preservatives		phenoxyethanol

### Evaluation items and measurements

The primary endpoint was determining bacterial count in the subgingival plaque, as measured by quantitative polymerase chain reaction (qPCR). Subgingival plaque from three teeth with 4–6 mm periodontal pockets was collected using paper points [32]. Total bacterial and *Porphyromonas gingivalis* (*P. gingivalis*: Pg) counts were determined by real-time qPCR using the extracted DNA samples as templates, according to methods established in our research laboratory [33].

As secondary endpoints, clinical examination involved measuring the periodontal pocket depth (PPD), bleeding on probing (BOP), and conducting a salivary analysis using the Salivary Multi-Test (SMT; LION Dental Products Company Limited, Tokyo, Japan). The PPD and BOP were assessed at three locations where subgingival margin plaque was collected by JSP Periodontists and JSP Board-Certified Periodontists (Japanese Society of Periodontology) using a PerioProbe #5 (YDM Corporation, Tokyo, Japan). The SMT kit was used according to the manufacturer’s instructions [34], and the six factors–cariogenic bacteria count, acidity, buffer capacity, leukocyte esterase, protein, and ammonia–were measured by collecting the discharge after rinsing the mouth with 3 mL of mouthwash for 10 s, applying 10 µL onto the test paper, and measuring for 5 min afterward with a dedicated instrument. The counts of cariogenic bacteria were assessed by detecting the reducing ability of resazurin in a group of gram-positive bacteria. The acidity related to dentin demineralization was evaluated by measuring the amount of hydrogen ions, as indicated by a color change in the pH indicator. The neutralizing capacity of saliva against acids was also analyzed (buffer capacity). Leukocyte esterase activity and total protein content in saliva were measured using the urine test paper technique. Ammonia, which is responsible for halitosis and other unpleasant odors, was identified using bromocresol green coloration.

### Statistical analysis

Comparisons of the participants’ backgrounds and laboratory values between the two groups were analyzed using the Mann–Whitney *U* test. Sex ratios were assessed using Pearson’s chi-squared test. Comparisons at baseline and after 4 weeks were evaluated using the Wilcoxon signed-rank test. In these analyses, statistical significance was set at *P* < 0.05. Statistical software JMP version 9.0.2 (SAS Institute Inc., Cary, NC, USA) was used for data analysis.

## Results

### The target sample of the study

**Fig. 1:**
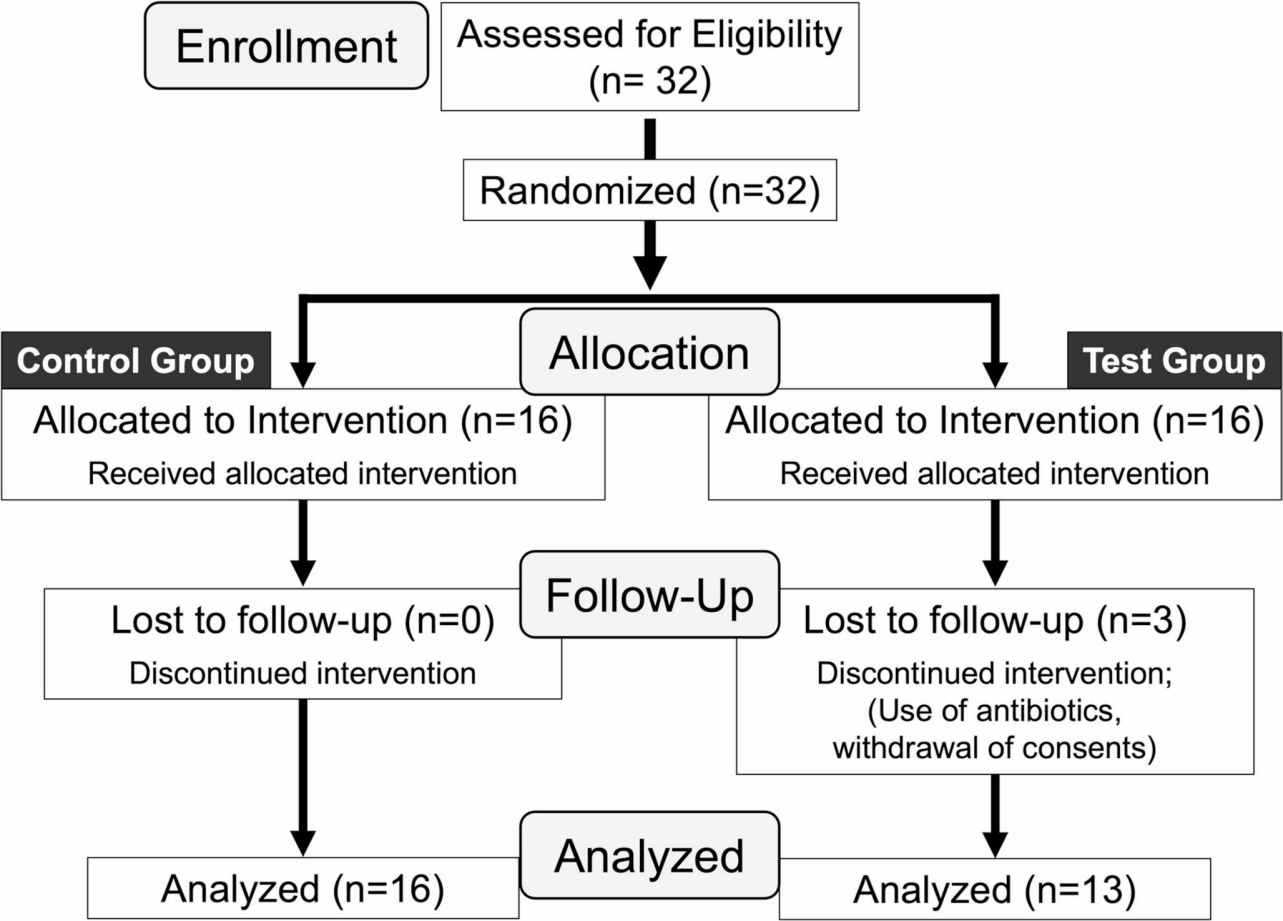
CONSORT flow diagram

A total of thirty-two Japanese patients (16 from each group) participated in the study conducted between February 1 and July 31, 2017. However, data from 29 patients (13 in the test group and 16 in the placebo group) were analyzed (Fig. 1). Three patients in the test group withdrew after obtaining informed consent but before clinical data could be collected because of the use of antibiotics for medical reasons and the withdrawal of consent for family circumstances. Patient characteristics are presented in Table 2. The age range was from 26 to 87 years old. The patients visit at intervals of two months or more (almost of them visit at three-month intervals). They keep SPT phase for an average of more than 30 months. At baseline, the *P. gingivalis* bacterial count was significantly lower in the test group; however, the other factors did not significantly differ between the two groups (Table 2). No adverse events were reported by any of the participants during the study period.

**Table 2:** Characteristics and parameters of baseline subject

	Test group (*n* = 13)	Placebo group (*n* = 16)	*P*-value
Sex	Male 3 Female 10	Male 4 Female 12	0.904
Age	65.3 ± 17.3	66.2 ± 12.7	0.881
Number of remaining teeth	25.6 ± 0.75	24.9 ± 0.67	0.469
Period from the start of SPT (month)	33.0 ± 45.8	54.4 ± 55.2	0.272
Total bacteria count (×10^8^)	2.08 ± 3.28	5.02 ± 5.88	0.136
*Pg* bacteria count (×10^8^)	0.14 ± 0.40	3.98 ± 1.11	0.028^*^
Periodontal pocket depth (mm)	4.56 ± 0.46	4.76 ± 0.93	0.825
Bleeding on probing (%)	52.7 ± 25.7	44.9 ± 23.7	0.414
Cariogenic bacteria count	33.8 ± 29.1	42.3 ± 28.1	0.482
Acidity	55.7 ± 24.3	68.2 ± 21.9	0.160
Buffer capacity	50.0 ± 27.6	36.9 ± 19.6	0.236
Leukocyte esterase	58.2 ± 22.6	57.3 ± 20.2	0.844
Protein	58.1 ± 24.5	58.0 ± 24.1	0.878
Ammonia	69.8 ± 23.8	58.5 ± 28.3	0.300

Data are expressed as mean ± standard deviation; comparisons between the two groups were analyzed with the Mann-Whitney U test

The sex ratio was analyzed by Fisher’s Exact test

At baseline, a significant difference in *Pg* bacteria count was observed between the two groups (*P < 0.05)

*Pg*: *Porphyromonas gingivalis*, SPT: supportive periodontal therapy

### Changes in bacterial counts

**Fig. 2:**
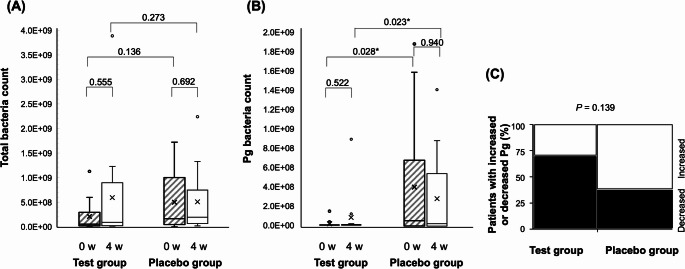
Changes in bacterial numbers after propolis toothpaste. **(A)** Number of total bacteria, **(B)** Number of *Pg*, **(C)** Marimekko Chart of patients with increased or decreased *Pg* (%). Comparisons between the test (*n* = 13) and placebo (*n* = 16) groups are performed using the Mann–Whitney *U* test. Comparisons between values at 0 and 4 weeks are performed using the Wilcoxon signed-rank test. Comparison between patients with increased or decreased *Pg* in the Marimekko Chart is performed using the Fisher’s exact test (two-tailed test). The number of *Pg* in periodontal pockets is significantly lower in the test group than in the placebo group. (*P < 0.05). *Pg*: *Porphyromonas gingivalis*

The mean and median total bacterial counts increased slightly after four weeks in both the test and placebo groups, with no significant difference between the two groups (Fig. 2A). The mean and median *P. gingivalis* bacterial counts in the test group were significantly lower at both baseline and after four weeks than those in the placebo group (*P* = 0.028 and 0.023; Fig. 2B). Further analysis using the Marimekko Chart to compare the numbers of patients with increased or decreased *P. gingivalis* counts showed that the patient numbers in the test group decreased more than those in the placebo group (not significant; Fig. 2C).

### Changes in the PPD and BOP

**Fig. 3:**
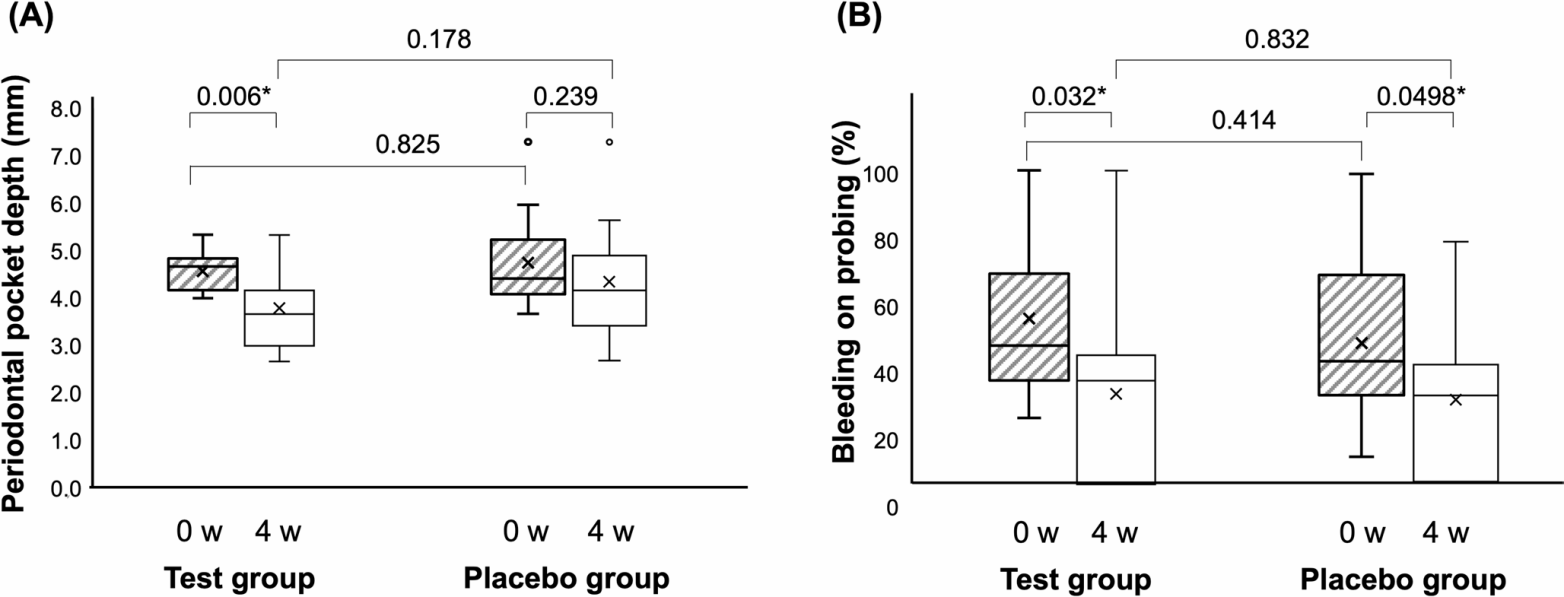
Changes in periodontal pocket depth (PPD) and bleeding on probing (BOP). Comparisons between the test (*n* = 13) and placebo (*n* = 16) groups are performed using the Mann–Whitney U test. Comparisons between values at 0 and 4 weeks are performed using the Wilcoxon signed-rank test. (A) PPD is significantly reduced in the test group after 4 w. (*P < 0.05). (B) BOP is significantly reduced in the test and placebo groups after 4 w. (*P < 0.05)

PPD decreased after four weeks in both groups, with a significant reduction in the test group (*P* = 0.006; Fig. 3A). The mean pocket depth value after four weeks was lower in the test group (3.80 mm) than that in the placebo group (4.35 mm). However, no significant differences were observed between the two groups. The rate of BOP was significantly reduced after four weeks in both groups (*P* = 0.032 for the test group, *P* = 0.0498 for the placebo group; Fig. 3B). However, there was no significant difference between the two groups.

### Changes in salivary parameters

**Fig. 4:**
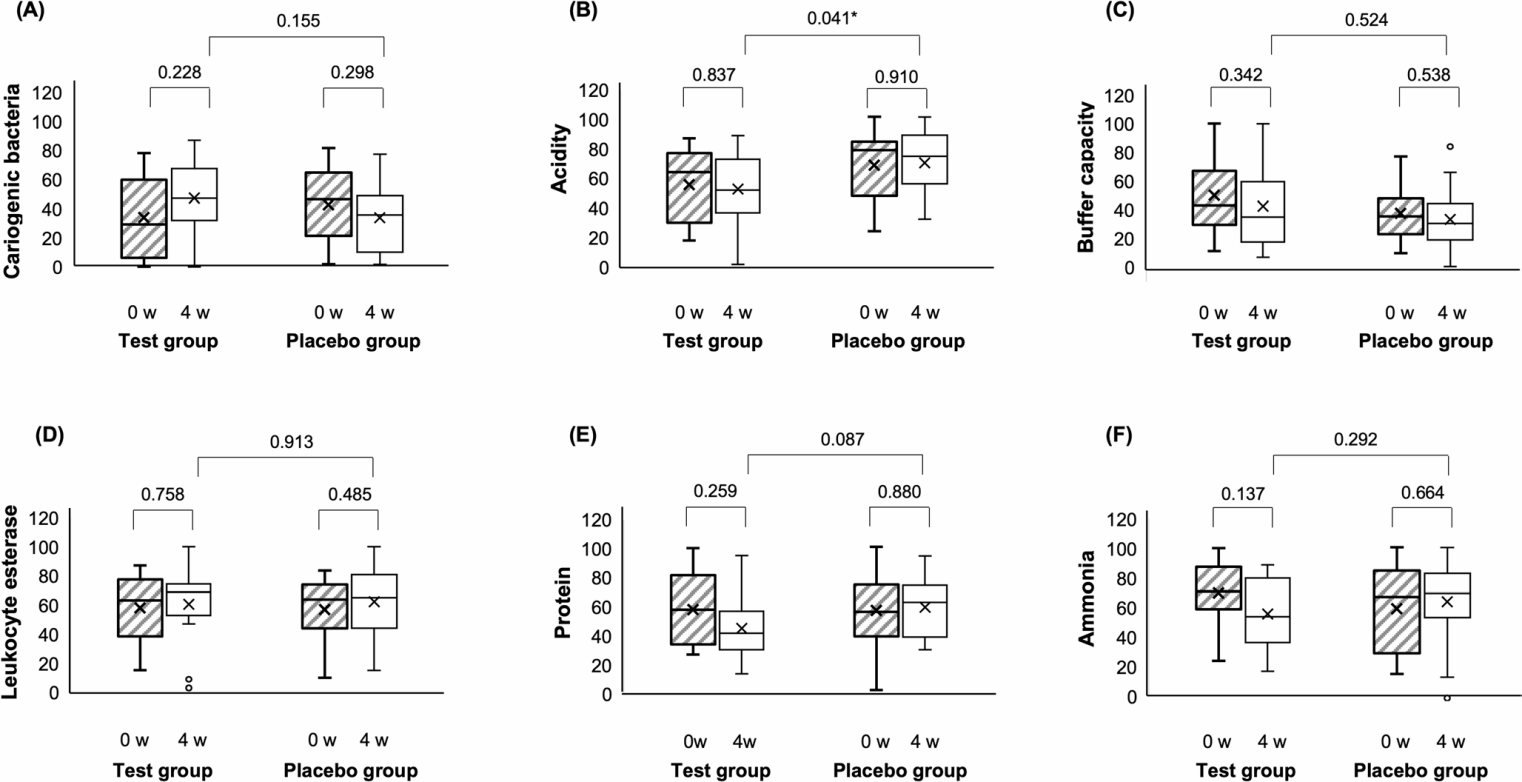
Changes in the test values of discharged saliva. (**A**): Cariogenic bacteria; (**B**): Acidity; (**C**): Buffer capacity; (**D**): Leukocyte esterase; (**E**): Protein; (**F**): Ammonia. Comparisons between the test (*n* = 13) and placebo (*n* = 16) groups are performed using the Mann–Whitney U test. Comparisons between values at 0 and 4 weeks are performed using the Wilcoxon signed-rank test. At 0 week, the two groups showed no significant differences in any parameters. Acidity at 4 weeks is significantly lower in the test group than in the placebo group (*P < 0.05). The test values are shown as indices obtained using the Salivary Multi-Test (LION)

The results of the six items assessed using the SMT are shown in Fig. 4. After four weeks, acidity was significantly lower in the test group than in the placebo group (*P* = 0.041; Fig. 4B). Protein levels were lower in the test group after four weeks (not significant, *P* = 0.087; Fig. 4E). The counts of other cariogenic bacteria, buffering capacity, leukocyte esterase, and ammonia levels did not change before and after the test or between the two groups.

## Discussion

In this randomized, double-blind clinical trial, we compared the safety and efficacy of propolis-containing toothpaste in patients with periodontitis under SPT. Participants were selected based on their status as patients with SPT who had completed a series of periodontal treatments and established self-care, making the effects of propolis on periodontal disease observable.

In the group that used toothpaste containing propolis for four weeks, the depth of periodontal pockets significantly decreased. In contrast, the placebo group, which used toothpaste without propolis, also showed a reduction in PPD. However, these changes were not statistically significant. The rate of BOP was also significantly reduced in both groups. However, these changes did not differ between groups (Fig. 3). Previous clinical trials have demonstrated significant improvements in clinical parameters such as PPD and BOP in groups using propolis [16, 20, 24]. This result aligns with previous findings, although relatively few studies have focused specifically on toothpastes containing propolis. During the study period, more thorough tooth brushing may have contributed to improvements in both groups. In this study, evaluations were limited locally to teeth with deep periodontal pockets; however, since the patients were in the SPT phase, it is likely that the periodontal inflamed surface area, which reflects inflammation throughout the entire oral cavity, remained stable.

No differences were observed in total bacterial counts or *P. gingivalis* counts within 4–6 mm periodontal pockets or in cariogenic bacterial counts in saliva. Amano et al. [24] analyzed the proportion of periodontal bacteria on the tongue surface after two weeks of using propolis-containing toothpaste among student volunteers aged 18–40 years. Their findings demonstrated a significant reduction in the counts of pathogenic bacteria, including (*P. gingivalis*), *Fusobacterium nucleatum* (*F. nucleatum*), and *Aggregatibacter actinomycetemcomitans* (*A. actinomycetemcomitans*), whereas the number of beneficial bacteria, such as *Streptococcus salivarius* (*S. salivarius*), remained unchanged. This suggests that propolis-containing toothpaste may effectively prevent periodontal disease. In this study, the *P. gingivalis*/total ratio was also assessed; however, the *P. gingivalis* counts in the propolis group were very low at baseline (Fig. 2B), which hindered confirmation of supportive results. Furthermore, several studies have shown significant reductions in *Streptococcus mutans* (*S. mutans*) and gram-positive bacteria [25, 35, 36], highlighting the inhibitory effects of propolis on the glucosyltransferase activity of these bacteria [25]. Because the SMT kit used in this study assessed the activity of gram-positive bacteria through resazurin reduction, it is possible that propolis did not reduce the number of cariogenic bacteria. Notably, there have been no reports on the interactions between propolis and resazurin. Another advantage of propolis is its potential to improve oral microflora [31, 37]. However, we must also be cautious about the shift or modification of oral microflora during long-term use [38].

In this study, the number of cariogenic bacteria in saliva increased, whereas oral acidity decreased after four weeks in the propolis group (Fig. 4AB). Acidity was significantly more substantial in the placebo group than in the propolis group at the four-week mark (a higher value of the Salivary Multi-Test means acidic). The exact pH values obtained from the SMT kit are unknown; however, the mean and median values in the placebo group were classified as “slightly high.” Propolis has been shown in diabetic rat studies to inhibit the acidification of interstitial fluid [39]. It may also prevent acidification in the mouth, bringing it closer to a neutral pH. In contrast, there were no obvious changes in buffer capacity, leukocyte esterase, protein, and ammonia (Fig. 4CDEF). This may be due to the selection of participants during a well-controlled SPT phase. These findings suggest that propolis improves the oral environment by regulating the acidity of the mouth.

Interestingly, propolis rinses have been reported to outperform chlorhexidine rinses in inhibiting *S. mutans* in the saliva of caries-active patients [40]. Similarly, propolis rinses have been shown to improve clinical parameters in perimenopausal women with periodontitis, comparable to those of chlorhexidine mouthwash [16]. Kodgi et al. [41] evaluated the in vitro antimicrobial activity of propolis in salivary samples from children with severe early childhood caries and found that propolis exhibited antimicrobial effects against both *S. mutans* and *Candida albicans* with minimal side effects. Therefore, propolis may be a viable alternative to chlorhexidine for long-term use. No adverse effects were observed in this study. While the prevalence of contact allergy to propolis was reported to be 3.6% in an earlier study [42], more recent clinical trials have not raised significant safety concerns, and no adverse effects, such as resin surface discoloration, have been noted over a four-year period [43]. Therefore, oral care products containing propolis validate the benefits of the natural remedies mentioned earlier.

However, this study has the following limitations. First, two limitations related to the lifestyle intervention are noted: the small sample size and the short intervention and observation period. We are fully aware of these two limitations. Due to the small sample size, many cases had low Pg bacteria counts at baseline, making it difficult to identify clear differences. Additionally, the lack of long-term follow-up after the 4-week observation period, despite continued SPT, is another limitation. Butera A, et al. [38] performed the clinical study for six months. However, they still described the 6-month follow-up as relatively short for evaluating the stability of clinical and microbiological improvements. Also, they noted that eventual low compliance or incorrect home use of the test products by the participants could have skewed the results. There may be no limits on the study periods, but longer periods are a potential risk for the quality of the study. In our study, selecting patients who had completed periodontal treatment and demonstrated good adherence to SPT and self-management allowed us to complete the clinical intervention study within the limited budget and timeframe, which was a strength of this study.

## Conclusion

In conclusion, propolis-containing toothpaste did not significantly affect the number of bacteria in the periodontal pockets or saliva of patients in the SPT phase with stable periodontitis. However, this led to a clinically significant reduction in the depth and bleeding of periodontal pockets. Furthermore, it is believed that the oral environment improved because of reduced salivary acidity. As aforementioned, the effects of propolis are mild; therefore, future research should involve large-scale intervention studies with more participants to assess the potential of propolis in oral care more accurately.

## Data Availability

No datasets were generated or analysed during the current study.

## Declarations

### Patient statement

Written informed consent was obtained from all participants after they were fully informed of the study procedure.

### Permission to reproduce material from other sources

All figures and tables in this article are original and have not been published in any other article.
